# Facilitation of synaptic transmission and pain responses by CGRP in the amygdala of normal rats

**DOI:** 10.1186/1744-8069-6-10

**Published:** 2010-02-08

**Authors:** Jeong S Han, Hita Adwanikar, Zhen Li, Guangchen Ji, Volker Neugebauer

**Affiliations:** 1 Department of Neuroscience & Cell Biology, The University of Texas Medical Branch, Galveston, Texas 77555-1069, USA

## Abstract

Calcitonin gene-related peptide (CGRP) plays an important role in peripheral and central sensitization. CGRP also is a key molecule in the spino-parabrachial-amygdaloid pain pathway. Blockade of CGRP1 receptors in the spinal cord or in the amygdala has antinociceptive effects in different pain models. Here we studied the electrophysiological mechanisms of behavioral effects of CGRP in the amygdala in normal animals without tissue injury.

Whole-cell patch-clamp recordings of neurons in the latero-capsular division of the central nucleus of the amygdala (CeLC) in rat brain slices showed that CGRP (100 nM) increased excitatory postsynaptic currents (EPSCs) at the parabrachio-amygdaloid (PB-CeLC) synapse, the exclusive source of CGRP in the amygdala. Consistent with a postsynaptic mechanism of action, CGRP increased amplitude, but not frequency, of miniature EPSCs and did not affect paired-pulse facilitation. CGRP also increased neuronal excitability. CGRP-induced synaptic facilitation was reversed by an NMDA receptor antagonist (AP5, 50 μM) or a PKA inhibitor (KT5720, 1 μM), but not by a PKC inhibitor (GF109203X, 1 μM). Stereotaxic administration of CGRP (10 μM, concentration in microdialysis probe) into the CeLC by microdialysis in awake rats increased audible and ultrasonic vocalizations and decreased hindlimb withdrawal thresholds. Behavioral effects of CGRP were largely blocked by KT5720 (100 μM) but not by GF109203X (100 μM).

The results show that CGRP in the amygdala exacerbates nocifensive and affective behavioral responses in normal animals through PKA- and NMDA receptor-dependent postsynaptic facilitation. Thus, increased CGRP levels in the amygdala might trigger pain in the absence of tissue injury.

## Introduction

Calcitonin gene-related peptide (CGRP) is a 37 amino acid peptide that binds to G-protein-coupled receptors, including CGRP1, which couple positively to adenylyl cyclase, cyclic AMP formation and protein kinase A (PKA) activation [1-4]. CGRP has emerged as an important molecule at different levels of the pain neuraxis [5]. Particularly high levels of CGRP binding sites [1,4,6,7] and proteins required for functional CGRP1 receptors [8,9] have been described in the superficial spinal dorsal horn and in the central nucleus of the amygdala (CeA), where also CGRP containing fibers terminate [4,7,10-13].

Work from our group and others showed that pain-related plasticity in the latero-capsular division of the CeA (CeLC) contributes critically to the emotional-affective dimension of pain [14,15]. The CeLC is essentially delineated by CGRP immunoreactive fibers that arise from the external lateral parabrachial area (PB) [12,13,16,17]. The PB projection to the CeLC provides purely nociceptive input to the amygdala as part of the spino-parabrachio-amygdaloid pain pathway that originates in lamina I [18-20]. Lamina I neurons projecting to the PB receive direct peptidergic afferent input [19], including from CGRP containing fibers [7].

Consistent with an important role of CGRP on the input and output sides of the spino-parabrachio-amygdaloid pain pathway, blockade of CGRP1 receptors in the spinal dorsal horn [21] or the amygdala [22] inhibited pain-related central sensitization of CeLC neurons and nocifensive and affective behaviors. Central sensitization of CeLC neurons involves synaptic plasticity at the PB-CeLC synapse and increased neuronal excitability [14,15] in models of arthritic [23], visceral [24] and neuropathic [25] pain. Arthritis pain-related plasticity in the CeLC depends on increased function of postsynaptic NR1/NR2B NMDA receptors through PKA-dependent NR1 phosphorylation [26-28] and endogenous activation of CGRP1 [22] and CRF1 [29,30] receptors. Mechanisms of pain-related PKA activation in the CeLC remained to be determined, but both CGRP1 and CRF1 receptors couple to PKA activation.

Central sensitization and plasticity in the CeLC correlate with increased pain behavior because pharmacologic deactivation of the CeLC with antagonists for group I metabotropic glutamate receptors [31,32], CGRP1 [22] and CRF1 [30] receptors, GABA-A agonist [33], and inhibitors of PKA, but not PKC, and ERK [34,35] decreased nocifensive and affective pain behaviors in different pain models. Conversely, in normal animals ERK activation in the CeLC [35], corticosterone delivery to the CeA [36] and block of GABA-A receptors in the CeA [33] increased peripheral hypersensitivity, although it was not clear if these effects correlated positively with amygdala activity.

Neuronal effects of CGRP in the amygdala remain to be determined. Mechanisms of CGRP actions in general are not well understood. In the spinal cord, CGRP increased responses of dorsal horn neurons [37-40] and nocifensive behavior [40-43] by increasing synaptic transmission and neuronal excitability [44,45]. Activation of PKA and PKC, modulation of AMPA and NMDA receptor function, and interactions with substance P have been implicated in the spinal actions of CGRP [5]. The goal of the present study was to determine synaptic and cellular effects of CGRP in the CeLC, underlying mechanisms and behavioral consequences in naïve animals without tissue injury.

## Methods

Male Sprague Dawley rats (150-350 g) were housed in a temperature controlled room and maintained on a 12 h day/night cycle. Water and food were available *ad libitum*. All experimental procedures were approved by the Institutional Animal Care and Use Committee (IACUC) at the University of Texas Medical Branch (UTMB) and conform to the guidelines of the International Association for the Study of Pain (IASP) and of the National Institutes of Health (NIH).

### Electrophysiology: patch-clamp recording

Coronal brain slices (300-500 μm) containing the CeLC were obtained from normal untreated rats (150-250 g) as previously described [22,30]. Rats were decapitated without the use of anesthesia to avoid chemical contamination of the tissue. A single brain slice was transferred to the recording chamber and submerged in artificial cerebrospinal fluid (ACSF; 31 ± 1°C), which superfused the slice at ~2 ml/min. ACSF contained (in mM) NaCl 117, KCl 4.7, NaH_2_PO_4 _1.2, CaCl_2 _2.5, MgCl_2 _1.2, NaHCO_3 _25, and glucose 11. The ACSF was oxygenated and equilibrated to pH 7.4 with a mixture of 95% O_2_/5% CO_2_. Only one or two brain slices per animal were used, one neuron was recorded in each slice, and a fresh slice was used for each new experimental protocol. Numbers in the manuscript refer to the number of neurons tested for each parameter.

Whole-cell patch-clamp recordings were obtained from CeLC neurons using the "blind" patch technique as previously described [22,30]. The boundaries of the different amygdalar nuclei are easily discerned under light microscopy (see Figure 1 in [30]). Recording pipettes (3-5 MΩ tip resistance) were made from borosilicate glass (1.5 mm and 1.12 mm, outer and inner diameter, respectively; Drummond, Broomall, PA) using a Flaming-Brown micropipette puller (P-80/PC, Sutter Instrument Co., Novato, CA). Electrodes were filled with intracellular solution containing (in mM): 122 K-gluconate, 5 NaCl, 0.3 CaCl_2_, 2 MgCl_2_, 1 EGTA, 10 HEPES, 5 Na_2_-ATP, and 0.4 Na_3_-GTP; pH was adjusted to 7.2-7.3 with KOH and osmolarity to 280 mOsm/kg with sucrose. Data acquisition and analysis of voltage and current signals were done using a dual 4-pole Bessel filter (Warner Instr.), low-noise Digidata 1322 interface (Axon Instr.), Axoclamp-2B amplifier (Axon Instr.), Pentium PC, and pClamp9 software (Axon Instr.). Signals were low-pass filtered at 1 kHz and digitized at 5 kHz. Headstage voltage was monitored continuously on an oscilloscope to ensure precise performance of the amplifier. High (> 2 GΩ) seal and low (< 20 MΩ) series resistances were checked throughout the experiment (using pClamp9 membrane test function) to ensure high-quality recordings. If series resistance (monitored with pClamp9 software, Axon Instr.) changed more than 10%, the neuron was discarded. Neurons were recorded at -60 mV.

**Figure 1:**
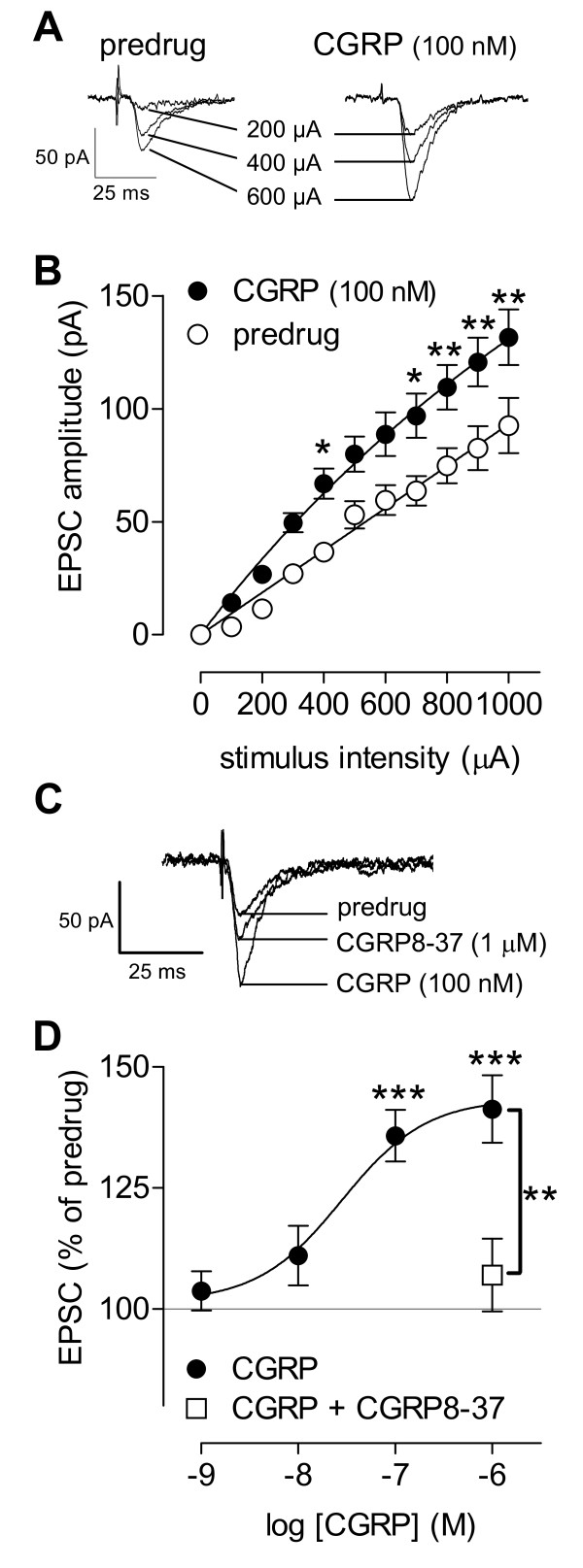
**CGRP enhances synaptic transmission in the CeLC in slices from normal animals**. **(A) **Monosynaptic EPSCs evoked at the PB-CeLC synapse with increasing stimulus intensities before and during CGRP (100 nM, 12 min). Individual traces are the average of 10 EPSCs. **(B) **CGRP (100 nM, 10-14 min) increased input-output function of the PB-CeLC synapse significantly (n = 10, P < 0.0001, F_1,198 _= 67.97, two-way ANOVA). Input-output curves were generated by plotting peak EPSC amplitude (pA) as a function of afferent fiber volley stimulus intensity (μA). **(C) **Synaptic facilitation by CGRP was blocked by co-administration of a CGRP1 receptor antagonist (CGRP8-37, 1 μM). Individual traces are the average of 8-10 EPSCs. **(D) **Cumulative concentration-response relationship of CGRP effects on synaptic transmission at the PB-CeLC synapse (n = 15). Peak amplitudes of monosynaptic EPSCs were averaged for each concentration of CGRP and expressed as percent of predrug control (set to 100%). Concentration-response curve was obtained by non-linear regression analysis using the formula *y *= *A*+(*B*-*A*)/[1+(10^*C*^/10^*X*^)^*D*^], where *A *is the bottom plateau, *B *top plateau, *C *= log(IC_50_), and *D *is the slope coefficient (GraphPad Prism software). CGRP8-37 (1 μM, n = 6) blocked the effect of CGRP (100 nM). CeLC neurons were recorded at -60 mV in slices from naïve untreated animals. Symbols and error bars represent mean ± SEM. *, **, *** P < 0.05-0.001 (Bonferroni posttests).

Using concentric bipolar stimulating electrodes (SNE-100, Kopf Instr.; 22 kW), monosynaptic EPSCs were evoked in CeLC neurons by focal electrical stimulation (Grass S88 stimulator) of inputs from the PB. For stimulation of the PB-CeLC synapse, the electrode was positioned under microscopic control on the afferent fiber tract from the lateral PB, which runs dorsomedial to the CeA and ventral to but outside of the caudate-putamen [22,30]. In the vicinity of this tract, no other afferents to the CeA have been described [17,46]. Electrical stimuli (150 μs square-wave pulses) were delivered at low frequencies (< 0.25 Hz). Input-output functions were obtained by increasing the stimulus intensity in 100 μA steps. For evaluation of a drug effect on synaptically evoked responses, the stimulus intensity was adjusted to 75-80% of the intensity required for orthodromic spike generation.

For paired-pulse ratio (PPR) analysis two orthodromic synaptic stimuli of equal intensity were applied at varying intervals and the resulting EPSCs were recorded. Peak amplitudes of the initial EPSC (EPSC1) and the second EPSC (EPSC2) were measured as the difference between the current level before the stimulus artifact and the peak of the EPSC. PPR was calculated as the ratio of EPSC2 over EPSC1 and expressed in %. Any alterations in PPR, a measure of short-term synaptic plasticity, suggest a presynaptic site of action [22,30].

Miniature EPSCs (mEPSCs) were recorded in TTX 1 μM as described previously [22,30]. A fixed length of traces (5 min) was analyzed for frequency and amplitude distributions using MiniAnalysis program 5.3 (Synaptosoft, Decatur, GA). The root mean square (RMS) of the background noise was computed for each set of data. The detection threshold for an event was set to three to four times the RMS value. Peaks were detected automatically, but each detected event was then visually inspected to prevent the inclusion of false data.

Drugs (see below) were applied by gravity-driven superfusion of the brain slice in the ACSF (~2 ml/min). Solution flow into the recording chamber (1 ml volume) was controlled with a three-way stopcock.

### Behavioral tests

Adult male Sprague-Dawley rats (250-350 g) were used in all experiments.

#### Spinal reflexes

Thresholds of hindlimb withdrawal reflexes evoked by mechanical stimulation of the knee joint were measured as described previously [47]. Mechanical stimuli of continuously increasing intensity were applied to the knee joint by means of a forceps equipped with a force transducer, whose calibrated output was amplified and displayed in grams on a liquid crystal display screen. Withdrawal threshold was defined as the minimum stimulus intensity that evoked a withdrawal reflex.

#### Vocalizations

Audible and ultrasonic vocalizations were recorded and analyzed as described previously [31,47,48]. The experimental setup (U.S. Patent 7,213,538) included a custom-designed recording chamber, a condenser microphone (audible range, 20 Hz to 16 kHz) connected to a preamplifier, an ultrasound detector (25 ± 4 kHz), filter and amplifier (UltraVox 4-channel system; Noldus Information Technology, Leesburg, VA), and data acquisition software (UltraVox 2.0; Noldus Information Technology), which automatically monitored the occurrence of vocalizations within user-defined frequencies and recorded number and duration of digitized events (vocalizations). Vocalizations in the audible and ultrasonic ranges were recorded simultaneously but with different microphones (condenser microphone and bat detector, respectively) connected to separate channels of the amplifier. The computerized recording system was set to suppress non-relevant audible sounds (background noise) and to ignore ultrasounds outside the defined frequency range. The chamber also had an opening for drug administration through the microdialysis probe inserted into the implanted guide cannula (see below). Animals were placed in the recording chamber for acclimation 1 h before the vocalization measurements.

Brief (15 s) innocuous (500 g/30 mm^2^) and noxious (2000 g/30 mm^2^) mechanical stimuli were applied to the knee, using a calibrated forceps (see above). Stimulus intensities of 100-500 g/30 mm^2 ^applied to the knee and other deep tissue are considered innocuous because they do not evoke hindlimb withdrawal reflexes in awake rats and are not felt to be painful when tested on the experimenters. Pressure stimuli >1500 g/30 mm^2 ^are noxious because they evoke hindlimb withdrawal reflexes in awake rats and are distinctly painful when applied to the experimenters [47]. The total duration of vocalizations (arithmetic sum of the duration of individual events) was recorded for 1 min, starting with the onset of the mechanical stimulus.

#### Drug application by microdialysis in awake animals

As described in detail previously [22,30,31,47], a guide cannula was implanted stereotaxically the day before behavioral measurements, using a stereotaxic apparatus (David Kopf Instr.). The animal was anesthetized with pentobarbital sodium (Nembutal^®^, 50 mg/kg, i.p.) and a small unilateral craniotomy was performed at the sutura frontoparietalis level. The guide cannula was implanted on the dorsal margin of the CeLC, using the following coordinates (in mm): CeLC, 2.0 caudal to bregma, 4.0 lateral to midline, depth 7.0. The cannula was fixed to the skull with dental acrylic (Plastics One, Roanoke, VA). Antibiotic ointment was applied to the exposed tissue to prevent infection. On the day of the experiment, a microdialysis probe (CMA12; CMA/Microdialysis Inc., North Chelmsford, MA; 20 kD cut-off, membrane length 2 mm) was inserted through the guide cannula so that the probe protruded beyond the tip of the guide cannula by 2 mm. The probe was connected to a Harvard infusion pump and perfused with ACSF (oxygenated and equilibrated to pH = 7.4). Before each drug application, ACSF was pumped through the fiber for at least 1 h to establish equilibrium in the tissue. Drugs were dissolved in ACSF on the day of the experiment at a concentration 100-fold that predicted to be needed based on data from our previous microdialysis and in vitro studies and data in the literature [5,22,30,45]. Drug concentration in the tissue is at least 100 times lower than in the microdialysis probe as a result of the concentration gradient across the dialysis membrane and diffusion in the tissue [22,30]. Numbers in the manuscript refer to drug concentrations in the microdialysis fiber.

#### Histological verification of drug administration sites

At the end of a behavioral experiment, the animal was sacrificed by decapitation using a guillotine (Harvard Apparatus Decapitator). This method of sacrifice is consistent with the recommendations of the Panel on Euthanasia of the American Veterinary Medical Association and approved by the Institutional Animal Care and Use Committee (IACUC). The brain was removed and submerged in 10% formalin. Tissues were stored in 20% sucrose before they were frozen sectioned at 50 μm. Sections were stained with Neutral Red, mounted on gel-coated slides, and coverslipped. Positions of the microdialysis fibers were identified under the microscope [30] and plotted on standard diagrams [from [49]].

### Drugs

Rat calcitonin gene-related peptide (**CGRP**) and **CGRP8-37 **(CGRP1 receptor antagonist) were purchased from Bachem, Torrance, CA. The following compounds were purchased from Tocris Bioscience (Ellisville, MO): (9R,10S,12S)-2,3,9,10,11,12-hexahydro-10-hydroxy-9-methyl-1-oxo-9,12-epoxy-1H-diindolo [1,2,3-fg:3',2',1'-kl]pyrrolo [3,4-i][1,6]benzodiazocine-10-carboxylic acid, hexyl ester (**KT5720**; membrane-permeable potent and selective **PKA **inhibitor [26,50]); 2- [1-(3-dimethylaminopropyl)indol-3-yl]-3-(indol-3-yl) maleimide (**GF109203x**; membrane-permeable potent and selective **PKC **inhibitor [51]); DL-2-amino-5-phosphonopentanoic acid (**AP5**; NMDA receptor antagonist). Drugs were dissolved in ACSF on the day of the experiment. ACSF served as vehicle control in all experiments.

### Statistical analysis

All averaged values are given as the mean ± SEM. Statistical significance was accepted at the level P < 0.05. GraphPad Prism 3.0 software (Graph-Pad Software, San Diego, CA) was used for all statistical analysis. For multiple comparisons, one-way ANOVA or two-way ANOVA was used with Bonferroni posttests to compare selected pairs of data). Paired student t-test was used to compare two sets of data that follow Gaussian distribution and have similar variances. Kolmogorov-Smirnov test was used for cumulative distribution analysis of mEPSCs (MiniAnalysis program 5.3 (Synaptosoft Inc., Decatur, GA). Concentration-response curves were obtained by non-linear regression analysis using the formula *y *= *A*+(*B*-*A*)/[1+(10^*C*^/10^*X*^)^*D*^], where *A *is the bottom plateau, *B *top plateau, *C *= log(IC_50_), and *D *is the slope coefficient (GraphPad Prism software).

## Results

Our previous studies showed that neurons in the latero-capsular part of the central nucleus of the amygdala (CeLC) develop synaptic plasticity and increased responsiveness in a model of arthritic pain [14,15] through a mechanism that involves endogenous CGRP1 receptor activation in the amygdala [22]. However, the effect of CGRP itself on synaptic transmission and neuronal excitability in the amygdala is not known and was determined in this study.

### Facilitation of synaptic transmission by CGRP

Whole-cell voltage-clamp recordings of CeLC neurons were made in brain slices from naïve untreated rats. Superfusion of the slices with CGRP significantly enhanced synaptic transmission, mimicking synaptic plasticity observed in the arthritic pain model. Monosynaptic EPSCs of progressively larger amplitudes were evoked by electrical synaptic stimulation of presumed PB afferents [30] with increasing intensities. Input-output relationships were obtained by measuring EPSC peak amplitude (pA) as a function of afferent fiber volley stimulus intensity (μA) for each neuron (Figure 1A). Baseline EPSCs are mediated by non-NMDA receptors as they persist in the presence of NMDA receptor blockade (see AP5 data below and our previous studies [26]). CGRP enhanced the input-output function of the PB-CeLC synapse significantly as evidenced by the steeper slope and upward shift at higher stimulus intensities (Figure 1B; n = 10, P < 0.0001, F_1,198 _= 67.97, two-way ANOVA). The facilitatory effects of CGRP were concentration-dependent (apparent EC_50 _= 28.3 nM; Figure 1D; n = 15, P < 0.001, F = 9.681, one-way ANOVA). A CGRP1 receptor antagonist (CGRP8-37, 1 μM) blocked the facilitatory effects of CGRP (100 nM) significantly (n = 6; P < 0.01, one-way ANOVA with Bonferroni posttest; see individual example in Figure 1C and averaged data in Figure 1D). CGRP1 receptor antagonists have no effect on baseline synaptic transmission in CeLC neurons [22]. These data show the presence of functional CGRP receptors in the CeLC under normal conditions.

### Post- rather than pre-synaptic site of action

**Figure 2:**
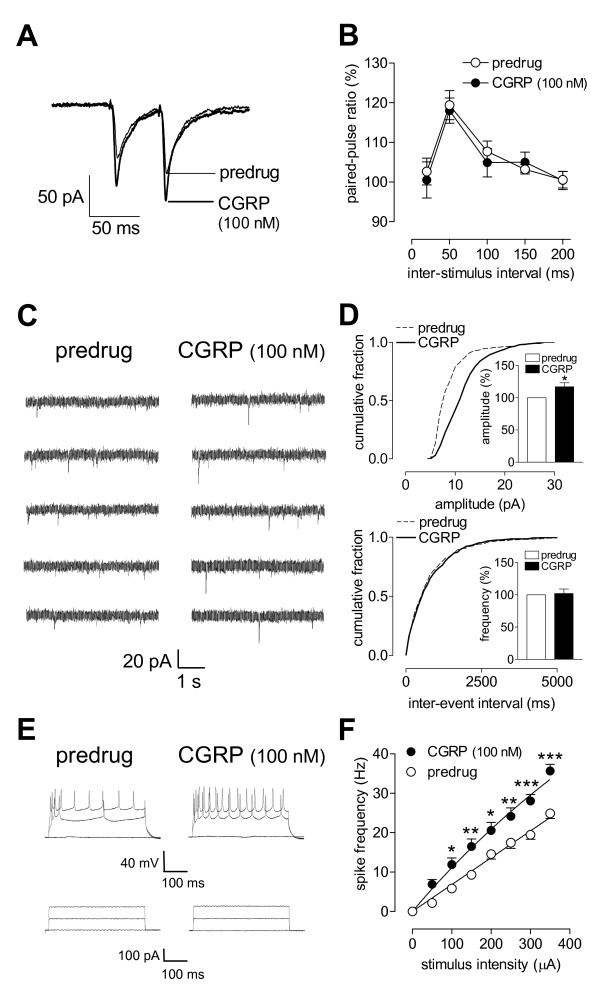
**Post- rather than pre-synaptic effects of CGRP**. **(A, B) **Paired-pulse ratio (PPR), a measure of presynaptic mechanisms, was not affected by CGRP (100 nM, 12 min). **(A) **Current traces (average of 8-10 EPSCs) recorded in an individual CeLC. Inter-stimulus interval, 50 ms. **(B) **CGRP had no significant effect on PPR in the sample of neurons (n = 12, P > 0.05, F_1,110 _= 0.24, two-way ANOVA). **(C) **Original current traces of mEPSCs recorded in an individual CeLC neuron in the presence of TTX (1 μM). CGRP (100 nM, 12 min) increased amplitude, but not frequency, of mEPSCs. **(D) **Cumulative distribution analysis of mEPSCs amplitude and frequency. CGRP (100 nM, 12 min) caused a significant shift toward larger amplitudes (n = 4, P < 0.001, Kolmogorov-Smirnov test) but had no effect on inter-event interval distribution. CGRP selectively increased mean mEPSC amplitude (P < 0.05, paired t-test) but not mean frequency (n = 4; see bar histograms showing data normalized to predrug control). **(E) **Number of action potentials evoked in a CeLC neuron by direct intracellular injections of depolarizing current pulses (500 ms) of increasing magnitude (lower traces) increased during superfusion of CGRP (100 nM, 12 min; upper traces). **(F) **CGRP increased input-output functions significantly (n = 11, P < 0.0001, F_1,156 _= 82.12, two-way ANOVA). Recordings were made in slices from naïve (untreated) animals. Neurons were recorded at -60 mV. Symbols and error bars represent mean ± SEM. * P < 0.05 (paired-test).

To determine whether CGRP acts pre- or post-synaptically in the CeLC we used a number of well-established electrophysiological methods, including paired-pulse facilitation (PPR) and miniature EPSC (mEPSC) analysis. These parameters were measured before and during application of CGRP. PPR was calculated as the ratio of the second and the first of two consecutive EPSCs evoked at the PB-CeLC synapse by two electrical stimuli of equal intensity at increasing inter-stimulus intervals (Figure 2A, individual example; Figure 2B, summarized data). CGRP (100 nM, 12 min) had no significant effect on PPR at various inter-stimulus intervals (n = 12, P > 0.05, F_1,110 _= 0.24, two-way ANOVA), arguing against a presynaptic action. The analysis of amplitude and frequency distribution of mEPSCs in the presence of TTX can be used to determine pre- versus post-synaptic mechanisms. Presynaptic changes at the transmitter release site affect mEPSC frequency, whereas changes at the postsynaptic membrane alter mEPSC amplitude (quantal size) [22,30,52]. CGRP (100 nM, 12 min) increased the amplitude, but not frequency, of mEPSCs recorded in CeLC neurons in the presence of TTX (1 μM) (Figure 2C; n = 4). CGRP caused a significant shift of the cumulative mEPSC amplitude distribution towards larger amplitudes (P < 0.001, Kolmogorov-Smirnov test; Figure 2D) and increased the mean mEPSC amplitude significantly (P < 0.05, paired t-test; Figure 2D, bar histogram). CGRP had no effect on the frequency of mEPSCs (see cumulative inter-event interval distribution, P > 0.05, Kolmogorov-Smirnov test; mean mEPSC frequency, P > 0.05, paired t-test; Figure 2D). The results of PPR and mEPSC analysis are consistent with a postsynaptic site of action of CGRP.

To determine the effect of CGRP on neuronal excitability, action potentials were evoked in current-clamp mode by direct intracellular current injections of increasing magnitude through the patch electrode. Input-output functions of neuronal excitability were obtained by averaging the frequency of action potentials evoked at each current intensity. Neurons were regular-spiking and showed no accommodation of action potential firing in response to sustained depolarization, which is characteristic of Type A projection neurons [53,54]. CGRP significantly increased the input-output function of CeLC neurons (Figure 2E, F, n = 11, P < 0.0001, F_1,156 _= 82.12, two-way ANOVA).

### Inhibition of PKA, but not PKC, blocks CGRP-induced synaptic facilitation

**Figure 3:**
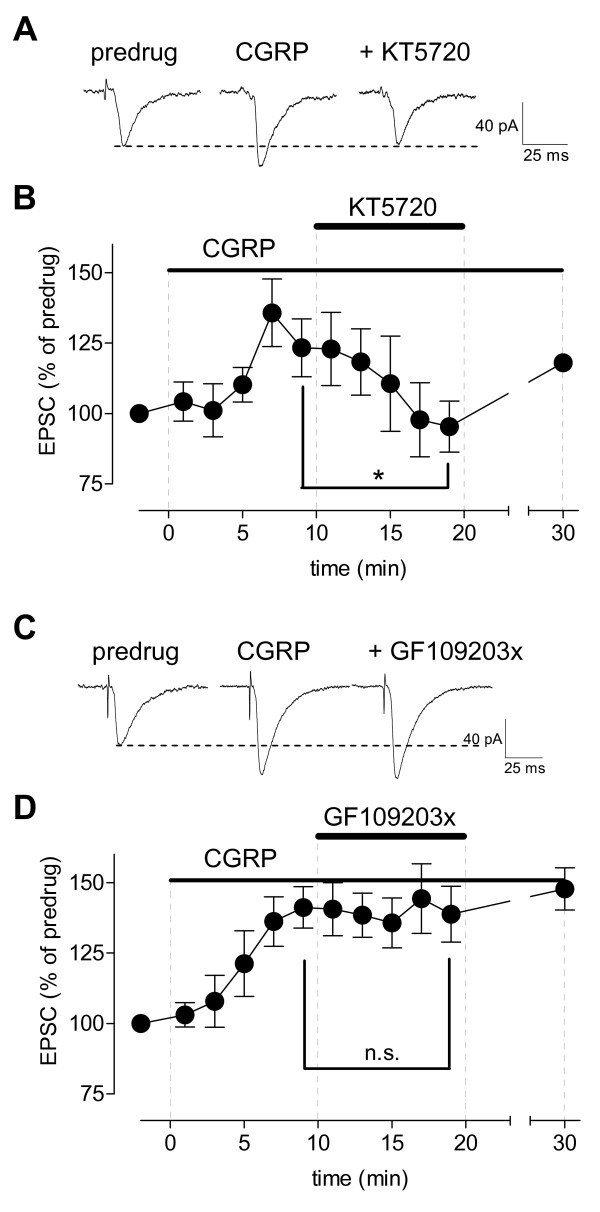
**Inhibition of PKA, but not PKC, blocks synaptic effects of CGRP**. **(A) **Original recordings of monosynaptic EPSCs (average of 10 EPSCs) evoked at the PB-CeLC synapse. Facilitatory effects of CGRP (100 nM) were blocked by co-administration of a PKA inhibitor (KT5720, 1 μM). **(B) **Summary of time course data for the sample of CeLC neurons (n = 7) show the inhibitory effects of KT5720 were reversible after washout. Peak amplitudes of EPSCs recorded during drug application were expressed as percent of predrug control values (set to 100%). **(C) **Individual traces (average of 8-10) of monosynaptic EPSCs show that the facilitatory effects of CGRP (100 nM) were not blocked by co-administration of a PKC inhibitor (GF109203x, 1 μM). **(D) **Summary of time course data for the sample of CeLC neurons show that the effects of CGRP did not desensitize during drug application for 30 min (n = 6; display as in **(B)**). Symbols and error bars represent mean ± SEM. * P < 0.05; n.s. (not significant), P > 0.05 (paired t-test, comparing the last measurement before and during application of cAMP-RP or KT5720). Statistical analysis was performed on raw data.

PKA, but not PKC, plays a critical role in pain-related plasticity in the CeLC [34]. The mechanism of PKA activation is not clear yet, but CGRP receptors couple to cAMP formation and PKA activation [1-4]. Therefore, we tested the hypothesis that CGRP-induced synaptic facilitation depends on PKA. A selective PKA inhibitor (KT5720, 1 μM) decreased synaptic facilitation by CGRP significantly (Figure 3A, B, n = 7, P < 0.05, paired t-test compared to predrug). The effect of KT5720 was reversible. In contrast, a selective PKC inhibitor (GF109203x, 1 μM) had no significant effect on CGRP-induced synaptic facilitation (Figure 3C, D, n = 6, P > 0.05, paired t-test).

### CGRP-induced synaptic facilitation involves NMDA receptors

**Figure 4:**
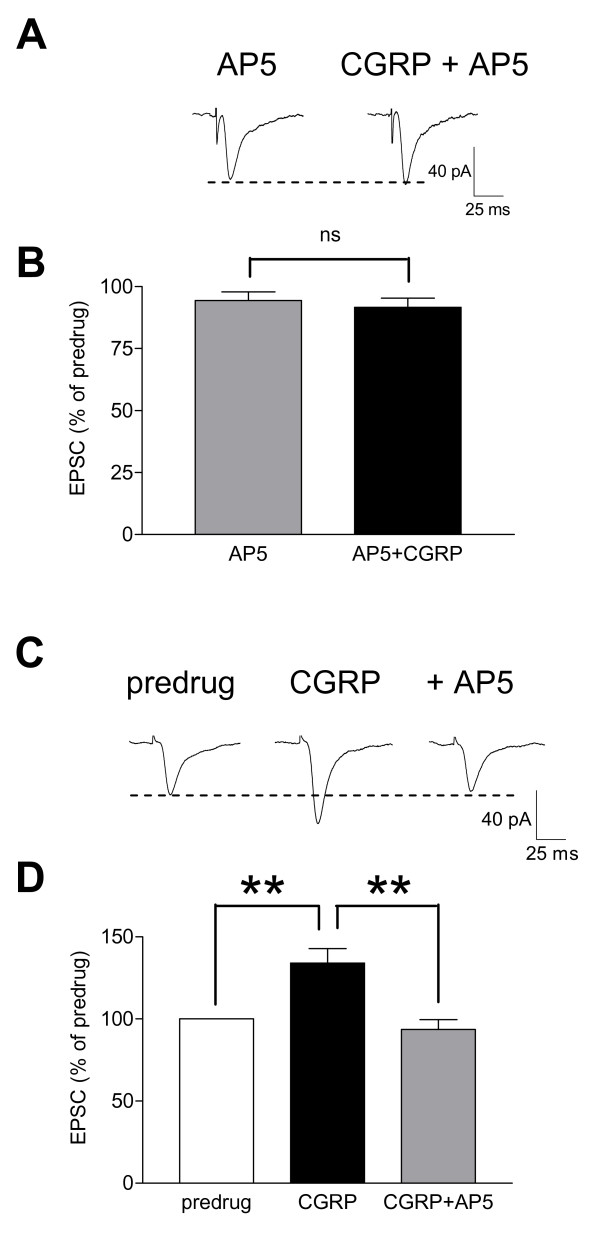
**NMDA receptor antagonist blocks CGRP effects**. **(A) **Original recordings of monosynaptic EPSCs (average of 8-10 EPSCs) evoked at the PB-CeLC synapse in the presence of AP5 (50 μM). CGRP (100 nM) had no effect. **(B) **Normalized data for the sample of CeLC neurons (n = 4). Peak amplitudes of EPSCs recorded during drug application were expressed as percent of predrug control values (set to 100%). **(C) **Individual traces (average of 8-10) of monosynaptic EPSCs show that the facilitatory effects of CGRP (100 nM) were blocked by co-administration of AP5 (50 μM). **(D) **Normalized data for the sample of CeLC neurons (n = 4; display as in **(B)**). Bar histograms show mean ± SEM. n.s. (not significant), P > 0.05 (paired t-test), ** P < 0.01 (ANOVA with Bonferroni posttests). Statistical analysis was performed on raw data.

PKA-dependent increase of NMDA receptor function is an important mechanism of arthritis pain-related synaptic plasticity in the CeLC [26]. Here we sought to determine if CGRP-induced synaptic facilitation also depends on NMDA receptors. Baseline synaptic transmission at the PB-CeLC synapse is mediated by non-NMDA receptors [26]. In the presence of an NMDA receptor antagonist (AP5, 50 μM) CGPR had no significant effect on synaptic transmission (Figure 4A, B, n = 4, P > 0.05, paired t-test). Likewise, AP5 (50 μM) inhibited CGRP-induced synaptic facilitation significantly (Figure 4C, D, n = 4, P < 0.01, one-way ANOVA with Bonferroni posttest). The data suggest that synaptic facilitation by CGRP depends on NMDA receptors rendered functional at resting membrane potentials as previously described in the arthritis pain model [26].

### Inhibition of PKA, but not PKC, blocks CGRP-induced behavioral responses

The behavioral consequences of pain-related changes in the CeLC include increased vocalizations in the audible and ultrasonic ranges and increased spinal reflexes [14,22,30,31,34], which are inhibited by blockade of CGRP1 receptors in the CeLC [22]. Here we determined the effects of CGRP administration into the CeLC on spinally (hindlimb withdrawal reflexes) and supraspinally (vocalizations) organized behaviors of normal naïve animals.

#### Vocalizations

**Figure 5:**
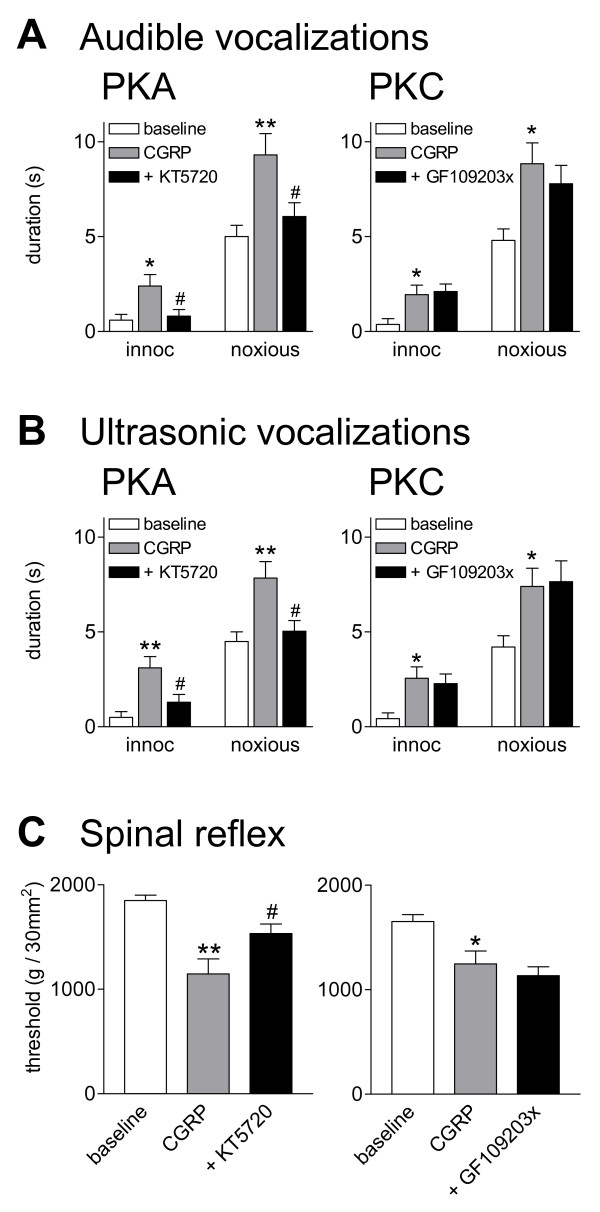
**Inhibition of PKA, but not PKC, blocks behavioral effects of CGRP**. Vocalizations and hindlimb withdrawal thresholds were measured in awake rats before and during application of CGRP into the CeLC. Audible **(A) **and ultrasonic **(B) **vocalizations in response to brief (15 s) compression of the knee with innocuous (500 g/30 mm^2^) or noxious (2000 g/30 mm^2^) intensity (see Methods) were measured for 1 min starting with the onset of the stimulus (see Methods). No vocalizations were detected in a control period of 5-10 min before stimulation. Administration of CGRP (10 μM, concentration in microdialysis probe; 15 min) into the CeLC evoked or increased vocalizations of naïve rats (n = 5 in each group). Co-administration of a PKA inhibitor (KT5720, 100 μM, n = 5) reversed the effects of CGRP; a PKC inhibitor (GF109203x, 100 μM, n = 5) had no significant effect. **(C) **Thresholds of hindlimb withdrawal reflexes measured by compressing the knee with a calibrated forceps (see Methods) were decreased by CGRP administered into the CeLC (10 μM, concentration in microdialysis probe; 15-20 min; n = 5 in each group). Co-administration of KT5720 (100 μM, n = 5) partially reversed the effects of CGRP. GF109203x (100 μM, n = 5) had no significant effect. Bar histograms and error bars represent mean ± SE. * P < 0.05, ** P < 0.01 (ANOVA with Bonferroni posttests, compared to predrug control). ^# ^P < 0.05 (ANOVA with Bonferroni posttests, compared to CGRP).

Audible vocalizations evoked by an aversive stimulus represent a supraspinally organized nocifensive response whereas ultrasonic vocalizations reflect the affective state of the animal [47,48]. Vocalizations were measured using a computerized analysis system as described previously [22,31,47] (see Methods). Vocalizations in the audible (20 Hz to 16 kHz; Figure 5A) and ultrasonic (25 ± 4 kHz; Figure 5B) ranges were evoked by brief innocuous (500 g/30 mm^2^) and noxious (2000 g/30 mm^2^) stimulation of the knee. Vocalizations were recorded for a period of 1 min starting with the onset of the stimulus. No apparently different effects were found in this study on vocalizations during stimulation and vocalization afterdischarges [31]. Therefore, the total duration (sum of individual vocalization events) is shown. Rats did not vocalize spontaneously in a control period of 5-10 min before stimulation. Administration of CGRP (10 μM, concentration in the microdialysis probe; 15 min) into the CeLC increased the duration of audible (Figure 5A) and ultrasonic (Figure 5B) vocalizations significantly (n = 5 in each group, P < 0.05-0.01 compared to predrug controls, Bonferroni posttests). CGRP increased vocalizations to innocuous and noxious stimuli, which is consistent with the presence of allodynia and hyperalgesia, respectively [48]. Predrug baseline measurements were made during administration of ACSF through the microdialysis probe as vehicle control.

Co-administration of a PKA inhibitor (KT5720, 100 μM, concentration in microdialysis probe) reversed the effects of CGRP on audible (Figure 5A) and ultrasonic (Figure 5B) vocalizations significantly (n = 5, P < 0.05 compared to predrug baseline, Bonferroni posttests). In contrast, co-administration of a PKC inhibitor (GF109203x, 100 μM) had no significant effect on CGRP-evoked vocalizations (Figure 5A, B, n = 5). In a separate group of rats the effect of CGRP was reversed by CGRP8-37 (100 μM, concentration in the microdialysis probe; n = 4, P < 0.05 compared to CGRP, Bonferroni posttests; data not shown).

#### Spinal reflexes

Thresholds for hindlimb withdrawal reflexes were determined by compressing the knee joint with gradually increasing stimulus intensities using a calibrated forceps whose output was displayed on an LCD screen (see Methods). Application of CGRP (10 μM, 15-20 min) into the CeLC decreased reflex thresholds significantly (Figure 5C, n = 5 in each group, P < 0.05-0.01, compared to predrug baseline, Bonferroni posttests), reflecting allodynic pain behavior. Co-administration of a PKA inhibitor (KT5720, 100 μM) reversed the effects of CGRP on (Figure 5C, n = 5, P < 0.05 compared to predrug baseline, Bonferroni posttests), whereas a PKC inhibitor (GF109203x, 100 μM) had no significant effect (Figure 5C, n = 5). The effect of CGRP was reversed by CGRP8-37 (100 μM, concentration in the microdialysis probe; n = 4, P < 0.05 compared to CGRP, Bonferroni posttests; data not shown).

#### Histology and placement controls

**Figure 6:**
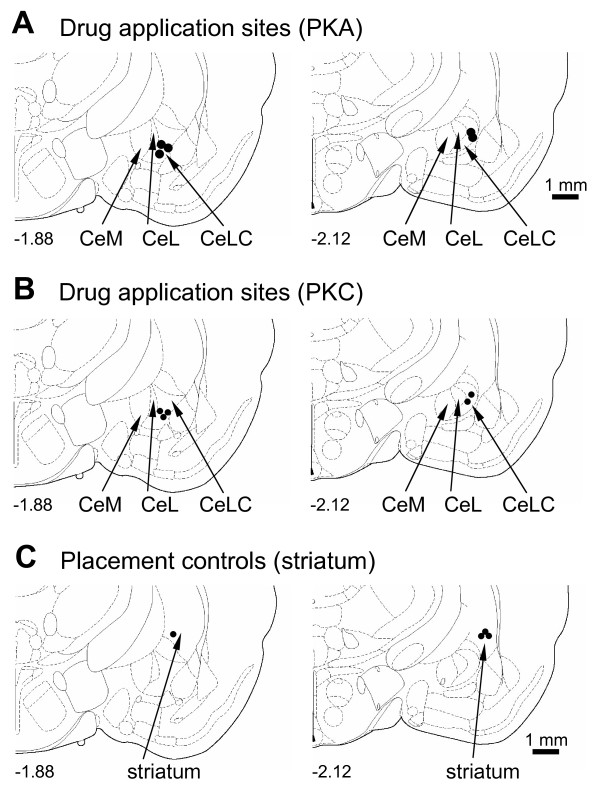
**Histological verification of drug application sites**. Diagrams adapted from [49] show coronal sections through the right hemisphere at different levels posterior to bregma (-1.88 and -2.12). Next to each diagram is shown in detail the CeA and its subdivisions, the medial (CeM), lateral (CeL) and latero-capsular (CeLC) part. Each symbol indicates the location of the tip of one microdialysis probe. The boundaries of the different amygdala nuclei are easily identified under the microscope (see Figure 1 in [30]). Calibration bars for diagrams are 1 mm.

Drug application sites into the CeLC were verified histologically. Figure 6 shows the position of the tips of the microdialysis probes. Applications of CGRP (100 nM, 15-20 min) into the striatum as a control for the potential spread of drugs (Figure 6C) did not produce significant changes of audible and ultrasonic vocalizations in naïve rats (n = 4, P > 0.05, paired t-test; data not shown).

## Discussion

This study determined the effects of a non-opioid neuropeptide (CGRP) in the amygdala, a brain area that has emerged as an important neural substrate for the emotional-affective component of pain [14,15]. The results show for the first time that CGRP in the amygdala (CeLC) of normal animals increases nocifensive and affective behaviors by increasing synaptic transmission and neuronal excitability. Synaptic facilitation results from a postsynaptic mechanism that involves PKA and NMDA receptors. The key findings are as follows: (1) Exogenous CGRP facilitated synaptic transmission at the PB-CeLC synapse that provides nociceptive information to the CeLC from the parabrachial area as part of the spino-parabrachio-amygdaloid pain pathway [18-20]. (2) Analysis of miniature EPSCs and paired-pulse facilitation indicates a post- rather than presynaptic action of CGRP on CeLC neurons. (3) CGRP also increased neuronal excitability of CeLC neurons, suggesting a direct cellular effect. (4) The effects of CGRP were largely blocked by a PKA, but not PKC, inhibitor and by an NMDA receptor antagonist. (5) Exogenous application of CGRP into the CeLC increased vocalizations and spinal reflex responses of normal naïve animals. (6) A PKA, but not PKC, inhibitor reversed CGRP-induced behavioral changes. (7) Electrophysiological and behavioral effects of CGRP were antagonized by CGRP8-37.

The significance of these findings is that increasing CGRP in the amygdala can produce or facilitate pain-like behaviors in normal animals and these behavioral effects correlate with increased neuronal activity. Pain arising from altered brain functions in the absence of tissue injury represents an important concept that may apply to so-called functional pain as well as pain states induced by trigger mechanisms such as in migraine. Central CGRP actions are thought to play a key role in migraine pathophysiology [2,55,56]. Other primary headaches and disease states such as temporomandibular disorders have been associated with elevated CGRP levels [55].

Particularly high levels of CGRP [4,10-13] and CGRP receptors [1,4,8,9] are found in the amygdala, a key player in affective states and disorders [57-60]. The amygdala is also involved in emotional-affective aspects of pain and in pain modulation [14,15]. Previous studies from our lab [22,26,29-31,34,61] and others [25,32,35] showed that increased amygdala activity in models of inflammatory and neuropathic pain correlated with increased pain behavior. It is conceivable that primarily non-pain-related activity changes in this brain area could affect pain responses and pain modulation. Affective disorders such as anxiety and depression modify activity in the amygdala [57,59,60] and are highly correlated with pain intensity and duration [62]. The present study demonstrates directly that increased amygdala activity in the absence of tissue injury or pain can exacerbate physiological pain responses such as withdrawal reflexes and vocalizations to "physiological" noxious stimuli (brief compression of peripheral tissue). This could be an important mechanism by which emotional disturbances gain access to pain modulation.

Pain-related changes in the CeLC include enhanced synaptic transmission and neuronal excitability through a mechanism that is centered on PKA, but not PKC, activation [34]. PKA increases synaptic transmission in the nociceptive parabrachio-amygdaloid pathway through phosphorylation of NMDA receptors [26]. Mechanisms of pain-related PKA activation in the CeLC are not entirely clear but our previous studies suggest that CGRP1 [22] and CRF1 [30] receptors could be upstream of PKA activation. Here we provide direct evidence that the facilitatory effects of CGRP in the CeLC involve PKA, but not PKC, and NMDA receptors. A PKA, but not PKC, inhibitor reversed the electrophysiological and behavioral effects of CGRP, and in the presence of an NMDA receptor antagonist CGRP had no effect. The reversibility of the effects of a PKA inhibitor, the persistence of facilitation in the presence of a PKC inhibitor, and the prevention of facilitation by pre-treatment with an NMDA receptor antagonist argue against de-sensitization of CGRP effects but support the involvement of PKA. In contrast, CGRP actions in the spinal cord involve both PKA and PKC [40] and modulate NMDA and AMPA receptor function [38,39]. Both PKA and PKC contribute to pain-related phosphorylation of NMDA receptors in the spinal cord [63,64] whereas only PKA is involved in the amygdala [26,34]. It remains to be determined if this discrepancy suggests different mechanisms of CGRP action at different levels of the pain neuraxis.

Interestingly, the kinetics of control and CGRP-evoked AP5-sensitive synaptic responses were largely similar, whereas NMDA receptors typically mediate slow EPSCs of longer duration [for recent review see [65]]. Further, AP5-sensitive EPSCs were recorded at a holding potential of -60 mV, where NMDA receptor channels would be expected to be blocked by magnesium. The data can be explained by the effects of receptor phosphorylation, which has been shown to relieve the magnesium block of NMDA receptors, rendering the channel functional even at -60 mV [66]. NMDA receptor phosphorylation by PKA or PKC also accelerates the rise and decay times of the ion channel [67,68], which would explain the absence of apparent differences in kinetics in the present study [for discussion see [34]].

The precise mechanism of action of CGRP is not fully understood, in part because of the complexity of the CGRP receptor(s). Functional CGRP1 receptors are formed by a heterodimeric complex of the calcitonin receptor-like receptor (CRLR) and receptor activity-modifying protein 1 (RAMP1) [69,70]. Overexpression of RAMP1 enhanced the pronociceptive effects of CGRP in the spinal cord [42]. CRLR and RAMP1 are expressed abundantly in cells of the amygdaloid complex, including the central nucleus [9], indicating the presence of functional CGRP1 receptors. However, CGRP can interact with receptors other than CGRP1 [3,69,71]. A CGRP1 receptor antagonist (CGRP8-37) did not antagonize the facilitatory effects of CGRP on AMPA-evoked responses of spinal dorsal neurons, which was interpreted as evidence for the involvement of a yet unknown receptor [38]. A CGRP2 receptor has long been postulated but its constituents have not been identified. Recently it was suggested that "CGRP2" receptors may not be a distinct molecular entity but an "amalgamation of contributions from a variety of CGRP-activated receptors" such as those that include RAMP2 or RAMP3 rather than RAMP1 [71]. In the amygdala, the BLA contains relatively more RAMP2 than RAMP1 whereas in the CeA RAMP1 expression is highest [9], consistent with the presence of CGRP1 receptors. Interestingly, anti-nociceptive effects of CGRP have been reported in the BLA of normal animals, but the synaptic and cellular actions were not determined [72]. CGRP receptor composition in different nuclei of the amygdala could explain the discrepancy with the present study. Activation of inhibitory projections from the BLA to the CeLC [54] may also account for inhibitory behavioral effects of CGRP in the BLA. Finally, mixed effects of CGRP could result from presynaptic or indirect network actions in addition to direct cellular effects. The neuronal effects of CGRP in the BLA remain to be determined.

The consequence of increase in synaptic transmission and excitability by CGRP was increased nocifensive and affective responses (withdrawal reflexes and audible and ultrasonic vocalizations) to brief physiological noxious stimuli in the absence of tissue injury. Behavioral effects of amygdala activation suggest that the recorded neurons have access to brainstem centers involved in pain modulation and generation of vocalizations. CeLC neurons recorded in this study had non-accommodating action potential firing properties. Non-accommodating cells, which represent the major neuronal type in the CeA, were identified previously as medium-size spine-laden Type A neurons [53,54]. Type A neurons are peptidergic or GABAergic projection neurons with targets in the brainstem, including PAG, and forebrain [53,73,74]. Peptidergic (CRF or enkephalin containing) CeA projection neurons are innervated by CGRP containing terminals [17,46]. Pain-facilitating effects of CGRP observed in this study could be due to activation of descending facilitatory brainstem centers or inhibition of descending inhibition [15,75]

## Conclusion

CGRP increases synaptic transmission and excitability in CeLC neurons in brain slices from untreated naïve animals, leading to increased spinally and supraspinally organized pain responses. Synaptic facilitation results from a postsynaptic mechanism that involves PKA and NMDA receptors. The study advances not only our knowledge of CGRP functions in the brain but also our understanding of pain as a complex condition that can be triggered and/or exacerbated by neuromodulator actions in the brain in the absence of tissue injury.

## Competing interests

The authors declare that they have no competing interests.

## Authors' contributions

J.H. and Z.L. performed patch-clamp recordings, analyzed electrophysiology data, and provided figures. J.H. wrote the first draft of the manuscript. J.H., H.A., and G.J. obtained and analyzed behavioral data and provided figures and results in abstract form. V.N. conceptualized the hypothesis, designed and supervised the experiments, directed the data analysis, and finalized the manuscript. All authors read and approved the manuscript.
